# Combining Standard Conventional Measures and Ecological Momentary Assessment of Depression, Anxiety and Coping Using Smartphone Application in Minor Stroke Population: A Longitudinal Study Protocol

**DOI:** 10.3389/fpsyg.2017.01172

**Published:** 2017-07-12

**Authors:** Camille Vansimaeys, Mathieu Zuber, Benjamin Pitrat, Claire Join-Lambert, Ruben Tamazyan, Wassim Farhat, Catherine Bungener

**Affiliations:** ^1^Laboratory of Psychopathology and Health Processes, Psychology Institute, Université Paris Descartes-Sorbonne Paris Cité Paris, France; ^2^Neurology and Neurovascular Department, Saint Joseph Hospital Group, Université Paris Descartes-Sorbonne Paris Cité Paris, France; ^3^Child and Adolescent Psychiatry Department, Robert Debré Hospital, Assistance Publique-Hôpitaux de Paris Paris, France

**Keywords:** ecological momentary assessment, mHealth app, smartphone app, minor stroke, anxiety, depression, coping, quality of life

## Abstract

**Context:** Stroke has several consequences on survivors’ daily life even for those who experience short-lasting neurological symptoms with no functional disability. Depression and anxiety are common psychological disorders occurring after a stroke. They affect long-term outcomes and quality of life but they are difficult to diagnose because of the neurobiological consequences of brain lesions. Current research priority is given to the improvement of the detection and prevention of those post-stroke psychological disorders. Although previous studies have brought promising perspectives, their designs based on retrospective tools involve some limits regarding their ecological validity. Ecological Momentary Assessment (EMA) is an alternative to conventional instruments that could be a key in research for understanding processes that underlined post-stroke depression and anxiety onset. We aim to evaluate the feasibility and validity of anxiety, depression and coping EMA for minor stroke patients.

**Methods:** Patients hospitalized in an Intensive Neuro-vascular Care Unit between April 2016 and January 2017 for a minor stroke is involved in a study based on an EMA methodology. We use a smartphone application in order to assess anxiety and depression symptoms and coping strategies four times a day during 1 week at three different times after stroke (hospital discharge, 2 and 4 months). Participants’ self-reports and clinician-rates of anxiety, depression and coping are collected simultaneously using conventional and standard instruments. Feasibility of the EMA method will be assessed considering the participation and compliance rate. Validity will be the assessed by comparing EMA and conventional self-report and clinician-rated measures.

**Discussion:** We expect this study to contribute to the development of EMA using smartphone in minor stroke population. EMA method offers promising research perspective in the assessment and understanding of post-stroke psychological disorders. The development of EMA in stroke population could lead to clinical implications such as remotely psychological follow-ups during early supported discharge.

**Trial registration:** European Clinical Trials Database Number 2014-A01937-40

## Introduction

### Background

Recent estimations (Murray et al., 2012; Lozano et al., 2013; Feigin et al., 2015) ranked stroke as the second cause of death and the third health-problem leading to disability and loss of life years worldwide and is considered as a major health priority in contemporary world. In 2013, the number of stroke survivors was estimated at more than 25 million (Feigin et al., 2015) and almost 11 million of them were between 20- and 64-years-old (Krishnamurthi et al., 2015).

Stroke is an acute unforeseen medical condition caused by an interruption of the blood supply to the brain (ischemia or hemorrhage) which causes damages to the tissue. Those damages are frequently related to neurological, motor and cognitive symptoms which can variate in the severity and disablement. Minor Stroke is defined as an actual cerebrovascular accident with mild and short-lasting neurological symptoms causing no functional disability (Fischer et al., 2010; Green and King, 2010). Prevalence of minor strokes has been estimated to 60% of overall strokes (Rothwell et al., 2004; Green and King, 2010).

#### Depression and Anxiety in Stroke Survivors

Psychological and affective disorders often occur after a stroke. The most common are depressive and anxious symptoms. Prevalence of depression ranges from approximately 20 to 40% (Barkercollo, 2007; De Wit et al., 2008; Ayerbe et al., 2011, 2013) and prevalence of anxiety is estimated between 18 and 32% in general stroke survivors’ population (Barkercollo, 2007; Ayerbe et al., 2014; Schöttke and Giabbiconi, 2015).

Several studies focused on depression after a stroke. In comparison, very few studies have been published considering specifically minor stroke patients while the prevalence of depression has been estimated at the same rates than in general stroke survivors populations (Altieri et al., 2012; Shi et al., 2015).

Not only it appears essential to identify and to treat post-stroke depression (PSD) and post-stroke-anxiety (PSA) because of their symptoms burden but also because of their negative impact on functional outcomes and mortality after stroke (Chemerinski et al., 2001; Naess et al., 2010; Bartoli et al., 2013).

#### Predictors of Post-stroke Depression and Anxiety

##### Symptomatic components of mental disorders

Diagnosis of post-stroke psychological disorders is scrambled because of physical, somatic, vegetative, autonomic and cognitive signs of stroke that can forge or hide depressive or anxious symptoms (Spalletta et al., 2005; McGinnes, 2009; Carota and Bogousslavsky, 2012; Idiaquez et al., 2015).

Specific and reliable symptoms are needed to be defined in order to improve PSD and PSA diagnoses and treatment. In general stroke populations (including patients with motor and cognitive impairment) symptoms such as sadness, crying behaviors, negative thoughts and emotional reactivity during the acute phase predict the later levels of depression, whereas symptoms such as fatigue, apathy and concentration difficulties do not (Carota et al., 2005; Sibon et al., 2012; Lerdal and Gay, 2013). It would be relevant to explore if similar patterns could be found in minor stroke survivors on one hand, and it is important to replicate such studies in regard of post-stroke anxiety on the other hand.

##### Coping strategies

Even after a minor stroke with no disabling impairment, stroke has an impact on individuals’ daily life (Adamit et al., 2015; Fride et al., 2015; Taule et al., 2015). Not only survivors have to deal with the emotional charge of having experienced a stroke but living after the accident involves many adjustments in life habits in the short, middle and long terms (Salter et al., 2008; Peoples et al., 2011). Those modifications of stroke survivors’ daily life and the way they deal with daily stroke-related consequences influence psychological outcomes (Darlington et al., 2007; Rozon and Rochette, 2015; Visser et al., 2015). Indeed, coping strategies have been identified as a reliable predictors of depression and anxiety after a stroke (Rochette et al., 2007; Kootker et al., 2016; Wei et al., 2016). Some ways to deal with stroke-related stress in daily life could be protective of negative psychological outcomes, whereas other strategies could be maladaptive and could lead to depression and anxiety. Nevertheless, there are needs for further investigation considering those questions especially in minor stroke population. Moreover it appears that previous studies could have some limitations considering their ecological validity and their applicability in real life.

#### Limits of Previous Studies and Perspectives for Further Research

The large majority of studies presented earlier are based on designs resorting to classical standard measures of depressive and anxious symptoms and coping strategies. But those conventional methods have been controversial because of recall biases implied by retrospective self-report assessments (Smyth and Stone, 2003). One question should be raised concerning the ecological validity of previous findings and models concerning PSD and PSA based on classical methods: is it what happens in real life? Does it reflect the real contextually and temporally dynamics of the processes and evolution of depression, anxiety and coping after a stroke?

The Ecological Momentary Assessment (EMA) is a methodological approach which aims to be specifically designed for the purpose of measuring phenomenon in their real specific ecological context of appearance. Based on the immediate or short term description of a phenomenon, EMA allows to study behaviors in their real daily life environment while limiting recall biases (Shiffman et al., 2008; Ebner-Priemer and Trull, 2009). Thus, EMA allows to study theoretical models in psychopathology by observing dynamic processes between multiple emotional, behavioral and cognitive phenomenon directly in their real context of appearance (Carpenter et al., 2016).

Ecological Momentary Assessment has been developed in the past three decades in the context of psychological and behavioral research for many conditions such as depression (Wenze and Miller, 2010; aan het Rot et al., 2012; Telford et al., 2012), anxiety (Walz et al., 2014), substance use (Shiffman, 2009; Morgenstern et al., 2014; Serre et al., 2015) or somatic diseases (Finan et al., 2008; Sibon et al., 2012; Bring et al., 2013; Kim et al., 2016).

There are still few studies reporting psychological EMA of stroke populations and all of those studies exclusively focused on depression (Lassalle-Lagadec et al., 2012; Sibon et al., 2012; Jean et al., 2013; Mazure et al., 2014). Previous results showed that the different daily symptoms of depression after stroke have specific evolution: somatic symptoms are less lasting than emotional and cognitive ones (Sibon et al., 2012). Depression was also related to specific daily life behaviors (Jean et al., 2013) and emotional reactivity to daily positive and negative events after stroke (Mazure et al., 2014).

### Objectives

This article aims to describe an innovative study design for the assessment and investigation during 4 months (following stroke), of minor stroke patients’ psychological health (depression, anxiety), coping strategies and quality of life combining classical standard scales and questionnaires with EMA using a smartphone application.

Primary objective is a methodological one. We aim to evaluate feasibility, acceptability, quality and reliability of data collected by EMA in minor stroke patients at different times and in different contexts during the first 4 months following a stroke.

Secondary objectives focus on studying psychopathological manifestations after stroke and the processes involved in their onset and evolution. Thus, we have several objectives considering these points: (1) to estimate the frequency and assess the evolution of anxiety and depression in minor stroke population, (2) to determine the risk factors and determinants of PSA and PSD in minor stroke patients and to study if coping is a reliable predictor, (3) to describe the clinical and psychosocial characteristics of depressed and anxious individuals after a minor stroke, (4) to describe symptomatic characteristics of PSD and PSA, (5) to study the sequences and dynamic processes between anxiety and depression symptoms, coping strategies and contextual and ecological factors at different times (during the first days, at 2 months and at 4 months) and contexts (hospitalization, discharge, return to work) after stroke.

## Methods And Analysis

### Selection of Participants

#### Inclusion, Non-inclusion and Exclusion Criteria

Participants are adults (over 18-years-old) hospitalized in the Neurovascular Intensive Care Unit of the Paris Saint-Joseph Hospital Group between April 2016 and January 2017 for a first ever ischemic or hemorrhagic minor stroke. Minor stroke is defined as 0–5 score at the NIHSS during hospitalization and at hospital discharge (Adams et al., 1999; Fischer et al., 2010; Spokoyny et al., 2015).

Patients with current heavy medical affections (no remitted cancer, neurodegenerative disease), dementia and/or psychotic disorder or other somatic condition which significantly impacts daily life, with severe aphasia or non-French speaking, visual or motor impairment and/or significant disability (Rankin score at 3 and more) are not included.

Patients who have a relapse of stroke during the study are excluded and partial data is included in the analysis.

#### Sample Size

Forty participants will be included in the study. According to previous research using EMA for psychological assessment, an acceptance rate between 75 and 90% is expected (Johnson et al., 2009a,b). Thus, we expect data from 30 to 36 participants to perform analysis.

### Design of the Study and Procedure during Hospitalization, at Discharge and during Follow-up

**FIGURE 1:**
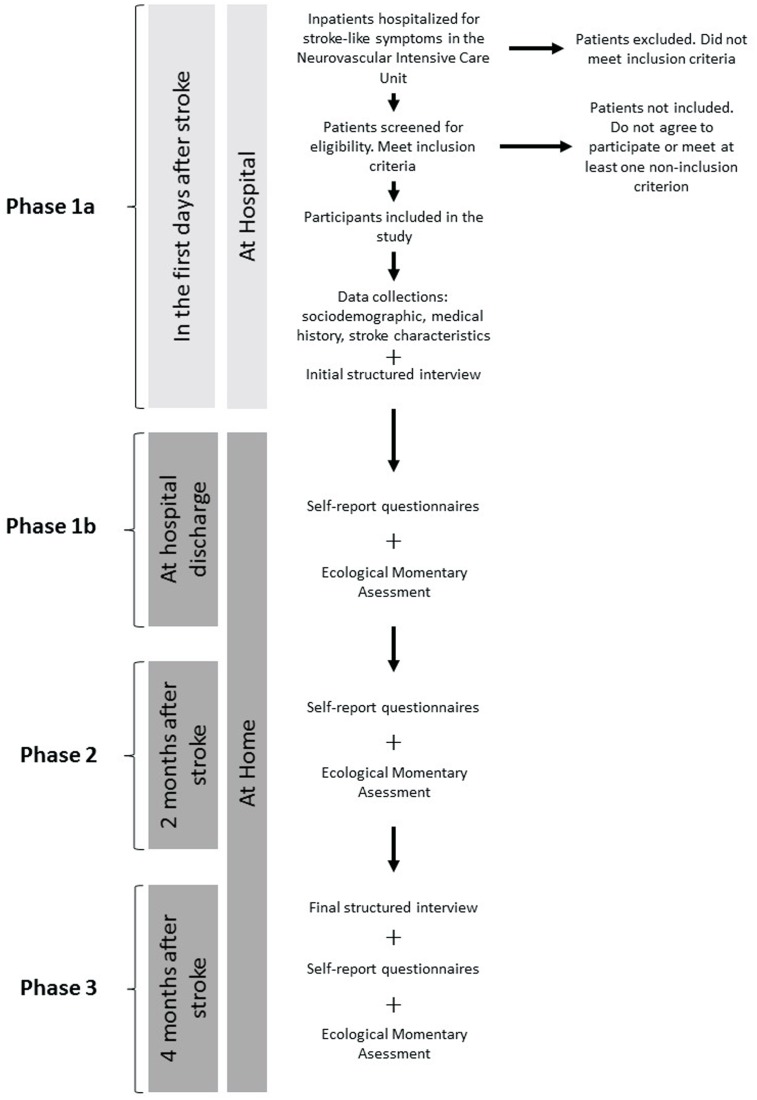
Flowchart of the study. Self-report questionnaires: assessment of depression and anxiety (BDI, HADS), quality of life (WHOQOL BREF) and coping strategies (Brief-COPE); Ecological Momentary Assessment: details of procedure and questions are available in Section “Material”; Structured interview: assessment of mood and anxiety symptoms and disorders (MINI, MADRS, HAM-A).

**Figure 1** presents the flowchart of the procedure of the study.

#### Phase 1

##### During hospitalization (phase 1a)

Each patient admitted to the Neurovascular Intensive Care Unit of the Paris Saint-Joseph Hospital Group for a stroke is screened by a physician for inclusion and non-inclusion criteria. The investigator, a psychologist, then meets eligible patients in order to explain the study and to give them an information letter containing the objectives and procedure. Patients who agree to participate sign the consent and then are assessed for depression and anxiety during a semi-structured interview.

##### At hospital discharge (phase 1b)

The psychologist investigator calls the patients to have an appointment in the first days following hospital discharge. Patients are met at home in order to start the EMA procedure. During this appointment, patients fill self-report questionnaires assessing depression, anxiety, quality of life and coping strategies with stroke related stress. After that, the investigator either installs the study application on patients’ personal devices or lent a device during the EMA procedure for patients who do not possess a smartphone. The content of the application is displayed and patients are trained to use it in order to resolve technical concerns and questions. After 2 days, patients are called by the investigator in order to check any problem and to reinforce the motivation to keep answering the prompts. At the end of the week, patients are informed by a phone call that they can disconnect from the app.

#### Phase 2: at 2-Months after Stroke

Patients are contacted by phone 2 months after stroke. They are invited to start the second phase of the study consisting in the same procedure as the one at phase 1b.

They answer EMA on the smartphone app during 1 week. By this time, we send them by mail the same self-report questionnaires and they send them back thanks to a pre-stamped envelope.

#### Phase 3: at 4-Months after Stroke

The psychologist investigator phones the patients 4 months after stroke in order to set an appointment at patients’ home for an interview during which depression and anxiety symptoms for the last 2 weeks are assessed. They fill the same self-report questionnaires. After the interview, the participants answer EMA on the smartphone during 1 week.

### Material

**Table 1** presents the assessment instruments used at each time of the study of each instrument.

**Table 1:** Variables measured in the study.

Variables	Administration	During hospitalization (Phase 1a)	At hospital discharge (Phase 1b)	2 months after stroke (Phase 2)	4 months after stroke (Phase 3)
**Psychiatric symptoms and disorders**					
MINI	Semi-structured interview	**X**			**X**
MADRS		**X**			**X**
HAM-A		**X**			**X**
BDI-II	Self-report questionnaires		**X**	**X**	**X**
HADS			**X**	**X**	**X**
EMA	Self-report on smartphone app		**X**	**X**	**X**
**Coping strategies**					
Brief-COPE	Self-report questionnaires		**X**	**X**	**X**
EMA	Self-report on smartphone app		**X**	**X**	**X**
**Quality of life**					
WHOQOL BREF	Self-report questionnaires		**X**	**X**	**X**
**Other measures**					
Sociodemographic, medical conditions and treatment, stroke characteristics and functional deficit	Clinician-report and medical file	**X**			

*MINI, Mini International Neuropsychiatric Interview; MADRS, Montgomery and Asberg Depression Rating Scale; HAM-A, Hamilton Anxiety rating scale; BDI-II, Beck Depression Inventory; HADS, Hospital Anxiety and Depression Scale; EMA, Ecological Momentary Assessment; Brief-COPE, Coping with Problem Experienced short version; WHOQOL BREF, World Health Organization Quality Of Life short version*.

#### EMA and Ad Scientiam Tool

In this study, the EMA is designed as a smartphone application: Ad Scientiam Research^®^. The app is programmed on participants’ personal (or lent) smartphones.

Each EMA phase (phase 1b = hospital discharge, phase 2 = 2 months post stroke and phase 3 = 4 months post stroke) lasts 1 week. The participants answer four brief electronic interviews (<3 min) per day during 7 days. The app sends prompts to inform the participants that they can answer the interview.

Time of prompts are fixed for each patient and randomized across patients. They occur within each of the following time periods: 7–10 am, 11 am to 2 pm, 3–6 pm and 7–10 pm. Each prompt is spaced out of at least 2 h from the prior and next prompts. A randomly generated daily schedule is submitted to participants. The time of prompts can be adjusted at each phase in order to suit participants’ typical daily schedules (wake, work, and sleep). The application permits responses to be provided within 30 min after each prompt.

The EMA consists in 32 screens related to seventeen questions split into three questionnaires (the exact wording is available in Supplementary File 1): (1) present situation and context; (2) anxiety and depression symptoms; (3) positive and negative events.

The selected items are based on previous EMA studies (Jean et al., 2013) or built specifically for the study on the basis of validated questionnaires. Questions composing questionnaire 2 have been adapted from the MINI (Major Depressive Episode and General Anxiety Disorder modules; Lecrubier et al., 1997; Sheehan et al., 1998). The anxiety and depression symptoms that can possibly variate within a day as it was previously done for an EMA study of depression in stroke population (Sibon et al., 2012) have been selected. For the questionnaire 3, questions about positive and negative events have been adapted from the Inventory of Small Life Events (Zautra et al., 1986) previously used and adapted for EMA (Petit, 2009; Mazure et al., 2014). Questions about the coping strategies for facing the negative event have been adapted from the Brief-COPE (Carver, 1997) and is an original EMA measure of coping created for the present study.

#### Content of EMA Questionnaires

Questionnaire 1 presents situation and context (multiple choice list): place, company, activity.

Questionnaire 2 – depression and anxiety symptoms (7 points Likert scale): sadness, anhedonia, fatigue, diminished concentration ability, negative thoughts on oneself, pessimism, anxiety, psychomotor agitation, physical tension, irritability.

Questionnaire 3 is related to positive and negative events that happened in the hours before the prompt: category of the most positive event and evaluation of the positive degree of this event on a 7-points Likert scale; category of the most negative event and evaluation of the negative degree of this event on a 7-points Likert scale; description of the reaction when confronted to this negative event. Fourteen sentences are answered by “yes” or “no” if they describe or not the behavior for coping with the situation.

Ecological Momentary Assessment questionnaires are explained and practiced with research staff at home when the first week of EMA begins. Then the procedure is reminded at the beginning of each week of EMA. All participants are contacted after 2 days during each EMA week to resolve any questions or difficulties.

#### Variables and Instruments

##### Psychiatric symptoms and disorders

*The Mini International Neuropsychiatric Interview* (MINI) (Sheehan et al., 1998) is a structured diagnostic interview used for DSM-IV (American Psychiatric Association, 2000) psychiatric disorders assessment. This is one of the most used instrument for psychiatric disorders diagnostic and it has been translated and validated in several languages including French (Lecrubier et al., 1997; Sheehan et al., 1998). It is composed of several modules corresponding with specific psychiatric disorders from DSM-IV. We use modules related to mood disorders (Major Depressive Episode, Dysthymia) and anxiety disorders relevant in the context of stroke (Post-traumatic Stress Disorders, General Anxiety Disorder).

*The Montgomery and Asberg Depression Rating Scale* (MADRS) (Montgomery and Asberg, 1979) is an observer-rated scale for depression symptoms assessment. It is composed of 10 items. Each one corresponds with a depressive symptom and is rated from 0 to 6 depending on the intensity of the symptom in the individual assessed. The scale has been validated in French (Lempérière et al., 1984). We refer to a structured interview guide for the administration of this scale which reinforce interrater reliability (*r* = 0.93) (Williams and Kobak, 2008). A cut-off point at 8 has been determined for screening depression after a stroke (Sagen et al., 2009; Kang et al., 2013).

*The Hamilton Anxiety Rating Scale* (HAM-A) (Hamilton, 1959) is an observer-rated scale for anxiety symptoms assessment composed of 14 items rated from 0 to 4. We use a French version of the scale previously validated (Pichot et al., 1981) and we refer to a structured interview guide for the administration of the scale in order to optimize interrater reliability (Shear et al., 2001). A cut-off point at 8 has been determined for screening anxiety in numerous previous research (Ballenger, 1999; Matza et al., 2010).

*The Beck Depression Inventory* (Beck et al., 1996) is a self-report questionnaire for depression symptoms composed of 21 items. Each item is composed of four statements corresponding to four level of intensity for the symptom. It is one of the most used instruments for the assessment of depression in clinical practice and research. A cut-off point at 11 has been used in previous studies in stroke population. This cut-off has a good sensitivity and specificity for the detection of depression after a stroke (Turner et al., 2012; Kang et al., 2013).

*The Hospital Anxiety and Depression Scale* (Zigmond and Snaith, 1983) is a 14-item self-report questionnaire designed to assess anxiety and depression severity. It is split into two scales of seven items: one scale for depression and one scale for anxiety. It is a widely used instrument in research. The scale has initially been developed for use in population with somatic conditions and it has good psychometric properties in several somatic populations and in stroke patients (Aben et al., 2002; Kootker et al., 2016). It presents the advantage of being a very short questionnaire while assessing symptoms on both somatic and psychological areas. Classically a score of 8 or more for each subscale is used as a cut-off score in studies to distinguish cases and non-cases of depression and anxiety. This cut-off point is also frequently used in stroke population and have a satisfying sensitivity and specificity in screening depression and anxiety disorders after a stroke (Sagen et al., 2009; Kang et al., 2013; Meader et al., 2014).

##### Coping strategies with stroke-related stress

Coping strategies are measured with the Brief-COPE (Coping with Problem Experienced short version) (Carver, 1997). The Brief-COPE is a 28-item self-report questionnaire divided in 14 subscales (two items by subscale) corresponding to conceptually differentiable coping reactions to stress: active coping, planning, instrumental social support, emotional social support, venting, positive reframing, acceptance, denial, self-blame, humor, religion, self-distraction, substance use and behavioral disengagement. We use a French validation of this instrument (Muller and Spitz, 2003). We refer to the dispositional version of the questionnaire and we have rephrased the wording of the instructions to focus on reactions concerning stroke-related stress. Each item is answered on a four-level scale corresponding to the frequency of the reaction when the individual is confronted to stroke-related stress situation.

##### Quality of life

We use the short version of the World Health Organization’s quality of life questionnaire (WHOQOL-BREF) for the dimensional assessment of quality of life (Bruesch et al., 2011). It is a 26-item self-report questionnaire measuring the subjective perception of the individual’s own life. The questionnaire concerns different fields: physical health (including among others pain, fatigue or working abilities), psychological health (including among other positive and negative emotions and self-esteem), social relations (including among other private life or the available social support) and living environment (including among other financial resources, access to information resources, recreation). Two introducing items are related to the overall quality of life. One score of specific quality of life is available for each domain and a general quality of life score is available by combining the specific scores. This instrument has previously been translated and validated in French (Leplège et al., 2000).

##### Other measures

###### Sociodemographic information

Including gender, age, marital status, familial situation at home (alone, spouse, child, etc), academic level, and socioeconomic status are collected or during the first interview either from the medical records.

###### Medical condition and treatment

Data about the medical condition of the participants including previous and present affections (somatic, neurologic, psychiatric), family history neurological affections, medical treatment at hospital entrance, vascular risk factors (BMI, diabetes, cholesterol, high blood pressure, alcohol and other substance use).

###### Stroke characteristics and functional deficit

Stroke localization (right or left hemisphere) and type (ischemic or hemorrhagic) based on medical imageries and exams conclusions. In case of ischemic stroke, we record if patients have received a thrombolytic treatment. Functional deficit is assessed with the National Institutes of Health Stroke Scale (NIHSS). This scale is administered by a trained health professional (physicians, nurses) and is composed of 15 items measuring neurological motor and cognitive deficits after a stroke. Items are related to consciousness, cognition, inattention, speech, sensory and motor functioning, visual fields functioning and ataxia (Meyer and Lyden, 2009; Zandieh et al., 2012).

#### Data Analysis

##### Outcomes and statistical methods for primary objectives

Feasibility and acceptability of the EMA method will be assessed by examination of acceptance and compliance rate, fatigue effects and reactivity to assessment: completion rate during each week, participation at follow-up weeks (phases 2 and 3), missing data and assessment duration across time during each phase. Descriptive analysis will be performed to describe feasibility and acceptability. Compliance will be assessed as the completion rate of EMA questionnaires. This rate will be calculated as the ratio of completed questionnaires to all sent questionnaires within each phase (four questionnaires per day during 7 days for each participant). Follow-up participation will be the ratio of number of participants at phase 2 and 3 to the number of participants at inclusion (phase 1). Fatigue effect will be calculated as the evolution of the within-day mean of missing data. Reactivity to EMA assessment will be estimated by comparing anxiety, depression and quality of life scores (self-reported questionnaires and clinicians’ ratings) of EMA participants to other participants (not involved in the EMA part of the study, more information is given in the limitations section below).

Validity of the method will be examined by using three different EMA indexes consisting in different aggregation of anxious and depressive symptoms assessed with EMA for each participant: a global anxiety and depression index (including all 11 symptoms assessed), a specific depression index (including sadness, anhedonia, fatigue, concentration difficulties, negative thoughts, pessimism) and a specific anxiety index (including anxiety, agitation, physical tension, irritability, concentration difficulties).

Validity will be assessed by using Pearson correlations between EMA indexes with self-reported questionnaires’ scores and clinicians’ ratings. Only participants with a satisfying minimum within- and between-day compliance will be included in this analysis (i.e., at least 5 EMA questionnaires filled). We will observe associations between: the global EMA index and the overall score of the HADS; the depression EMA index with the BDI score, the HADS depression subscale score and the MADRS score; the anxiety EMA index with the HADS anxiety subscale score and the HAM-A.

##### Outcomes and statistical methods for secondary objectives

(1) Frequency will be estimated as the ratio of depressed and anxious participants to the total number of participants. Presence of depression and anxiety will be determined in two different ways: according to the examination of psychiatric disorders during the semi-structured interview; and as a function of the cut-off scores of self-reported questionnaires and clinicians’ ratings scale previously reported in the instruments section.

(2) Univariate logistic regression analysis will be performed to study if the clinical variables (subtypes of anxiety and depression symptoms, coping strategies) predict depression and anxiety within each time and between times of the study.

(3) Descriptive analysis will be performed on clinical, sociodemographic and medical measures to describe depressed and anxious participants. Their results will be compared to healthy participants (presenting no significant depression and anxiety) with Chi-square and Student’s *t*-test.

(4) We will examine whether EMA of the different symptoms of depression and anxiety are associated with self-report and clinician-rated depression and anxiety to describe characteristics of PSD and PSA. Means-as-outcomes modeling will be used to describe those associations.

(5) In order to describe the time dynamics between anxiety and depression symptoms, coping strategies and contextual settings assessed with EMA, we will use a Multilevel Vector Autoregressive method (Bringmann et al., 2013). With the Vector Autoregressive (VAR) model, each variable at time point *t* is regressed to the time point *t* – 1 of every other variable. For example, sadness at 1 pm is regressed with all other depression and anxiety symptoms (including sadness) at 10 am. The multilevel-VAR model is suited to the hierarchical structure of EMA data and it enables to describe population dynamics as a function of within-person variability.

## Discussion

### Anticipated Results

#### Primary Objective

A participation rate at inclusion between 75 and 90% is expected regarding previous results with EMA method (Johnson et al., 2009a,b). The completion rate of EMA questionnaires is expected to be at least of 80% for the entire group during each phase (Sibon et al., 2012). In order to have satisfying validity, correlations between EMA indexes and data collected using other methods are expected to be higher than 0.70.

#### Secondary Objectives

We expect frequency of depression and anxiety in minor stroke population to be as high as frequency in general stroke. Indeed, even if the motor and cognitive impairments after a more severe stroke could lead to more severe anxiety and depression, previous study about prevalence of depression after minor stroke is in the range of prevalence found in general stroke population (Shi et al., 2015).

In line with previous results about PSD (Carota et al., 2005; Sibon et al., 2012; Lerdal and Gay, 2013), the results about the ecological characteristics of depression and anxiety after minor stroke could reveal different symptoms’ profiles as a function of time. Depression and anxiety at phase 1 would be characterized more by physical and non-specific symptoms such as fatigue, physical tension, attention difficulty whereas phase 2 and 3 profiles would have different profiles characterized by more specific symptoms.

Even if the investigation of time dynamics between depression and anxiety symptoms is greatly exploratory, we have similar expectations regarding the evolution of the importance and centrality of physical symptoms as function of time. Indeed, physical and non-specific would lead to more specific symptoms at phase 1 than at phase 2 and 3.

Results about coping strategies could follow the same pattern, with different adaptive and maladaptive coping at phase 1, 2, and 3. In regard of previous research (Darlington et al., 2007; Rochette et al., 2007), active coping would lead to less anxiety and depression at phase 1 than later.

### Research and Clinical Implications

#### Research Implications

Ecological Momentary Assessment using smartphone application is a wide area in terms of research perspectives. The great ecological validity of instantaneous capture of phenomenon in their real life contexts makes EMA method a promising perspective for research and knowledge in the field of psychopathology.

However, standard instruments still have to be developed. Recent published papers in different fields of psychology may be pieces of evidence that solid references are needed and that sharing protocols could help (Benarous et al., 2016; Mareva et al., 2016). We hopefully think that sharing and presenting the innovative protocol of the present work will contribute to develop EMA research. We expect this study to participate in the development of EMA research and methods in the area of psychological processes and more accurately in the study of populations with somatic and neurologic conditions.

The fact that we combined different assessment methods of psychopathology (i.e., self-report questionnaires, clinicians’ observation, EMA) is a main strength of this study. Indeed, the results will bring information regarding the validity of these different methods of psychological assessment.

Finally, the longitudinal EMA design of the study with three distinct phases at different times over a 4-month period is an original design for which feasibility has still to be informed.

#### Clinical Implications

Concerning the acute case of PSD and PSA etiopathogenesis, EMA research offers promising results so as to better understand the determinants, characteristics and the evolution of those emotional manifestations after a minor stroke. The EMA design of this study will help to clarify the biopsychosocial model of PSD and PSA (Aben and Verhey, 2006; Mast and Vedrody, 2006). Because of its particular ecological dimension, EMA makes possible to approach psychological phenomenon in real life environment. Thus, it enables to observe processes between behaviors, thoughts, emotions and environment. Our longitudinal EMA design could offers interesting results concerning those processes at different times after stroke.

We are expecting this study to contribute in the enhancement of diagnostic accuracy of psychiatric disorders and the detection of emotional distress in minor stroke population. We hopefully think the results of the present study could lead to identify specific reliable signs of depression and anxiety at different times after a stroke. Such results could help clinicians and care providers who work with stroke patients so as to facilitate the detection of emotional disorders by recommending them to focus on specific symptoms.

Finally, the remotely assessment of depression and anxiety using a smartphone application offers interesting perspectives for home-returned cares and follow-ups. Early supported discharge should be a better option for stroke patients (Mas and Inzitari, 2015) and is the main trajectory of minor stroke patients. The results of the present study will bring information concerning feasibility, validity and reliability of smartphone application psychological assessment after hospital discharge. Considering those results, further research could be suggested so as to develop a standard instrument for depression and anxiety screening after hospital discharge of stroke patients. Previous research in breast cancer population shown promising results in the field of mobile tool for screening depression (Kim et al., 2016). It still has to be developed in stroke population and it could lead to the improvement of the early supported discharge after a stroke by taking into account a remotely psychological follow-up.

### Limitations

The main limitation that could be mentioned is related to the respondent’s burden. It requires a substantial involvement for participants to answer EMA studies. Indeed, in our study there are three EMA phases during a period of 4 months, each one lasting 1 week. We are aware of this fact that is why we gave a major importance to make the study the less interfering and disturbing considering each participant schedule and daily life by using a quick smartphone electronic interview. Previous studies (Juengst et al., 2015; Maes et al., 2015) have shown that EMA participants well accept and are satisfied with this method.

Previous studies showing EMA in stroke population used five daily evaluations against only four in the present study (Lassalle-Lagadec et al., 2012; Sibon et al., 2012; Jean et al., 2013; Mazure et al., 2014). This main methodological difference is explained by the fact that previous research had no repeated EMA design. This reduction in the number of daily assessments has been chosen given the longitudinal perspective of the repeated EMA phases. Indeed, by reducing this number we expect to prevent a reduction in the participation at follow-up phases and so optimizing the quantity of data collected.

As it was previously underlined in studies focusing on methodological issues of EMA (Telford et al., 2012), answering to multiple daily psychological assessments should involve some reflection about biases and ethical concern. This issue is a general research issue known as the Hawthorne effect or the Participation to Research Effect (McCambridge et al., 2014a,b), but it could have a greater resonance considering EMA studies. Indeed, the repetitive confrontation to questions about negative daily life experiences could be considered as a double-edged sword: it forces people to remind and confront to bad experiences on one hand, but it can also make them more aware of their own experiences and needs so that they can eventually adjust to it (Shiffman, 2009; Telford et al., 2012; Runyan and Steinke, 2015).

We will give special concern to participants in the study and ethical requirements allow to help and to offer treatments for people who eventually will report mental distress and suffering during each phase.

Even the potential therapeutic effect of paying attention to self-feelings several times a day during EMA weeks could be a positive consequence for respondents, it could also be a significant bias of the study. However, the design of our study offers a possibility to test this bias. Indeed, we will include participants in the general study even if they refuse to participate to the smartphone application protocol. Those participants will not answer to EMA but they undergo the same exact procedure concerning questionnaires and interviews. We will then compare the evolution of psychological outcomes assessed by self-report questionnaires and clinician’s observation of the participants to the smartphone protocol to the individuals who refused to participate in this part of the study.

The compliance of participants is another important point that could impact the results because of the repeated daily assessment design of the study. This bias is a fatigue effect that have previously been studied in EMA studies in stroke population (Johnson et al., 2009b) and did not seemed to have a significant impact on participation as a function of study duration. Nevertheless, this study had no repeated design considering EMA. Thus, we will pay close attention to this potential effect in our analysis of feasibility. We described in the method section the particular implication of the investigator at each EMA phase so as to reinforce motivation of respondent and also the reduction of the number of daily prompts compared to previous studies so as to prevent the importance of potential fatigue effect because of the study design.

The point of the generalization of results also should be considered. The purpose of the study is to focus on mild stroke population given their particular health care compared to more severe stroke situations generally implying more medical concern and professional involvement. Nevertheless, the understanding of the pathogenesis of post-stroke mental disorders is important in both mild and severe stroke and ecological methods such as described in this article have been highlighted as promising perspective. It is clear that the present methods do not suit situations of more severe stroke with motor or cognitive impairments and that is why patient with motor or cognitive impairments are not included. Thus, the interpretation of results will hardly be generalizable to general stroke population. To date, the incompatibility with disability situations is one of the main limitations of EMA development in research. Current EMA instruments seem not to suit disability situations and so further research should design and evaluate suitable EMA methods for more severe. For example, daily repeated vocal recordings to automatically question could be used with motor dysfunction of upper limbs or visual difficulty after stroke instead of smartphone application with questions to read on different screens to scroll. Such designs would imply different experimental conditions and probably other biases that future study would have to take on.

## Ethics Statement

This study is carried out in accordance with the Comité de Protection des Personnes Ile-De-France II (CPP IDF II). All participants give their informed consent in accordance of the Declaration of Helsinki. As we mentioned earlier, a particular concern is given to patients’ safety. Participants will be informed and advised to share concerns with care professionals and their relatives whether they feel distress. Research staff will systematically contact participants eliciting psychological distress or suicidal ideation and direct them toward mental or medical care providers considering the participant’s needs. Participants will be instructed to answer prompts on the application only when it is safe and convenient. This protocol has already received ethical approval by the Comité de Protection des Personnes Ile-De-France II (CPP IDF II) and has been recorded on the European Clinical Trials Database (EudraCT Number/ID RCB 2014-A01937-40).

## Author Contributions

The study was originally design by CV, CB, and MZ. All of the authors further contributed to the research design, methodology, analysis plan and prospective discussion. CV drafted the first version of the manuscript and was assisted by CB, MZ, BP, CJ-L, RT and WF who contributed with critically revisions of intellectual contents. All authors approved the final manuscript.

## Conflict of Interest Statement

The authors declare that the research was conducted in the absence of any commercial or financial relationships that could be construed as a potential conflict of interest.
