# Supplementary material for: Combining Standard Conventional Measures and Ecological Momentary Assessment of Depression, Anxiety and Coping Using Smartphone Application in Minor Stroke Population: A Longitudinal Study Protocol
Source: Front Psychol. 2017 Jul 12;8:1172. doi: 10.3389/fpsyg.2017.01172 (PMC5506189; doi:10.3389/fpsyg.2017.01172)
Supplement: Supplementary file 1 [file EMA_questions.docx]

Supplementary File 1. Questions of EMA smartphone application

**Here is a new interview. Thank you for the time you give to bring responses!**

**Questionnaire 1**

***Where are you now?***

1 - At home

2 - At your relative’s place

3 - At your partner’s place

4 - At your friends’ place

5 - At work

6 - At a restaurant/a bar

7 - In a shop

8 - In a vehicle

9 - At hospital

10 - In an administration building

11 - In a garden

12 - Indoor (other)

13 - Outdoor (other)

***Whom are you with now?***

1 - Alone

2 - Partner

3 - Relatives

4 - Friends

5 - Co-workers

6 - Healthcare professional

7 - Unknown persons

8 - Pets

9 - Others: define afterward

***What is your main activity now?***

1 - Nothing

2 - Waiting

3 - Resting

4 - Eating

5 - Cooking

6 - Houseworking

7 - Working (payed)

8 - Shopping

9 - Hygienic care

10 - Discussion (face to face)

11 - Discussion (phone)

12 - Physical activty/sport

13 - Leisure

14 - Transportation

10 - Listening to music

11 - Watching TV

12 - Other: define afterward

**Questionnaire 2**

***To what degree are you feeling happy now?***

1 Not happy at all 2 3 4 5 6 7 Extremely happy

***To what degree are you feeling sad now?***

1 Not sad at all 2 3 4 5 6 7 Extremely sad

***To what degree are you feeling pleasure for what you are doing now?***

1 Neither interest nor pleasure 2 3 4 5 6 7 Much interest and pleasure

***To what degree are you feeling tired now?***

1 Not tired at all 2 3 4 5 6 7 Extremely tired

***To what degree are you focused on what you are doing now?***

1 Not focused at all 2 3 4 5 6 7 Completely focused

***Presently, to what degree do you agree with the sentence: “I am expecting*** ***more good things to happen to me than bad in the next hours”?***

1 Do not agree at all 2 3 4 5 6 7 Totally agree

***To what degree do you have negative thoughts about yourself now?***

1 No negative thought 2 3 4 5 6 7 Extremely negative thoughts

***To what degree are you feeling anxious now?***

1 Not anxious at all 2 3 4 5 6 7 Extremely anxious

***To what degree are feeling restless now?***

1 Totally quiet 2 3 4 5 6 7 Extremely restless

***To what degree are you feeling physical strain now?***

1 Totally relaxed 2 3 4 5 6 7 Extremely strained

***To what degree are you feeling irritable now?***

1 Not irritable at all 2 3 4 5 6 7 Extremely irritable

**Questionnaire 3**

***To which field does belong the most positive event that has happened since last prompt?***

1 - Family/marriage

2 - Work

3 - Recreation

4 - Home/household

5 - Health/illness related to stroke

6 - Health/illness not related to stroke

7 - Social life

8 - Legal matters

9 - Religion/spirituality

10 - Transportation

11 - Financial matters

12 - Others: define afterward

***To what degree has the event had a positive impact for you?***

1 No positive impact 2 3 4 5 6 7 Extremely positive impact

***To which field does belong the most negative event that has happened since last prompt?***

1 - Family/marriage

2 - Work

3 - Recreation

4 - Home/household

5 - Health/illness related to stroke

6 - Health/illness not related to stroke

7 - Social life

8 - Legal matters

9 - Religion/spirituality

10 - Transportation

11 - Financial matters

12 - Others: define afterward

***To what degree has the event had a negative impact for you?***

1 No negative impact 2 3 4 5 6 7 Extremely negative impact

***The following questions concern the way you have dealt with the NEGATIVE event you previously mentioned***

***I’ve been turning to work or other activities to take my mind off things***

YES

NO

***I’ve been using substances to make myself better (tobacco, alcohol, drugs, medication, food)***

YES

NO

***I’ve been getting emotional support from others***

YES

NO

***I’ve been giving up trying to deal with it***

YES

NO

***I’ve been trying to find comfort in my religion or spiritual beliefs***

YES

NO

***I’ve been trying to come up with a strategy about what to do***

YES

NO

***I’ve been making jokes about it***

YES

NO

***I’ve been expressing my negative feelings***

YES

NO

***I’ve been getting help and advice from other people***

YES

NO

***I’ve been concentrating my efforts on doing something about the situation I’m in***

YES

NO

***I’ve been doing as if it was not happening***

YES

NO

***I’ve been accepting the reality of the fact that it has happened***

YES

NO

***I’ve been blaming myself for things that happened***

YES

NO

***I’ve been looking for something good in what is happening***

YES

NO

**Thank you for your responses!**
